# Osteochondroma of the First Phalanx in Tbourida Horses

**DOI:** 10.3389/fvets.2018.00328

**Published:** 2019-01-08

**Authors:** Mohammed Seghrouchni, Enrico Bollo, Mohamed Piro, Hassan Alyakine, Hassan Bouayad, Jamal Chakir, Rahma Azrib, Khalid El Allali

**Affiliations:** ^1^Hassan II Institute of Agronomy and Veterinary, Veterinary University Hospital, Rabat, Morocco; ^2^Department of Veterinary Science, University of Torino, Turin, Italy; ^3^Department of Medicine Reproduction and Surgery, Hassan II Institute of Agronomy and Veterinary, Rabat, Morocco; ^4^The Royal Guard, Rabat, Morocco; ^5^Comparative Anatomy Unit, Department of Biological and Pharmaceutical Veterinary Sciences, Hassan II Institute of Agronomy and Veterinary, Rabat, Morocco

**Keywords:** osteochondroma, first phalanx, Tbourida, Arab Barb, horse

## Abstract

This study aimed at describing anatomo-histopathological and imaging features, using computed tomography and magnetic resonance imaging on six *ex vivo* forelimbs of Tbourida horses, that presented a particular bone exostosis on the dorsal and proximal part of the first phalanx, diagnosed by X-ray. Gross anatomy of the bone exostosis revealed an irregular surface with poly-lobulated tissue masses showing a cauliflower shape. The diameter/depth varied from 0.5 to 5.1 cm with a mean of 3.9 ± 0.9 cm. The capsule of the metacarpophalangeal joint was hypertrophic and showed many invaginations in the inner part, in contact with the bone exostosis. Computed tomography revealed cortical and medullary continuity of the bone exostosis, with the underlying bone, and remodeling of the cortical surface of the dorsal and proximal part of the first phalanx. Magnetic resonance imaging showed an increased signal intensity of the bone exostosis on the T1- and T2^*^-weighted gradient fast echo. Histological examination of the bone exostosis revealed a cap of hyaline cartilage, including large foci of endochondral ossification with a base of cancellous bone surrounding marrow spaces, which confirmed the diagnosis of osteochondroma. The capsule of the metacarpophalangeal joint showed a large amount of recently formed connective tissue fibers in its inner part, interspersed with mature connective tissue. The hyperextension of the metacarpophalangeal joint during a Tbourida show, which occurs on a hard ground surface, and the use of hobbles in horse stabling are most likely responsible for the outgrowth of an osteochondroma of different shapes and sizes, and fracture complications in some cases.

## Introduction

Horse sporting competitions have increased in popularity around the world. The horse industry underwent enormous progress over the last few last decades. As a consequence, there has been a rise in the frequency of horse injuries (1). This in turn has resulted in a high demand for veterinary assistance, to maintain the healthy state of athletic horses. It is well known that specific injuries are related to the category of sporting competitions and the performance levels of horses (2, 3). Indeed, some anatomical sites are more predisposed to the risk of specific injuries, depending on the sporting category horses take part in. Studies have shown that most injuries or diseases affecting the forelimbs, in quarter horses that participate in rodeo events, are navicular diseases and degenerative joint diseases (4). It is also known that flat race horses are more predisposed to the injury of the navicular region, while elite show jumping horses show a high risk of forelimb superficial digital flexor tendon and distal deep digital flexor tendon injuries (3).

Tbourida is a very popular traditional equestrian sport in North Africa, in which only Arab Barb horses are allowed to compete. In Morocco, this sport involves an important population of 2500 horses. A Tbourida show requires 15 riders, galloping for a distance of 200 m, unloading and reloading their muskets in different positions, throwing them in the air, and catching them before firing into the sky (5). Unlike some more traditional equestrian sports, Tbourida is quite demanding and requires sudden starts and stops. The stop is the finale, and representative of the physical demands of the Tbourida event. It is described as a sudden stop after a fast gallop, during which the horse engages his hind limbs under the body. This final conformation of the limbs is quite similar to that exhibited by the quarter horses used in rodeo shows. In Tbourida horses, the final sudden stop requires a hyperextension of the metacarpophalangeal joint (MCPJ).

Because of its angular design, which allows a wide range of movements, the MCPJ is known to be subjected to high biomechanical stresses. Alternations of hyperextension and extreme flexion of this joint, are responsible for tremendous tensive and torque forces on the fetlock soft tissues predisposed to lameness (6). In a previous radiographic screening study of osteoarticular diseases of the distal forelimbs of Tbourida horses conducted in Morocco, a particular bone overgrowth exostosis of the dorsal and proximal part of the first phalanx (P1) was diagnosed. This was observed in 20% of Tbourida horses and seems to have never been described in other breeds of equestrian horses. In this study, we aimed to undertake a focus study on this particular lesion on the dorsal and proximal part of the P1, in order to diagnose its exact pathological nature. Therefore, investigations included anatomo-histopathological analysis combined with the study of its imaging features using computed tomography (CT) and magnetic resonance imaging (MRI).

## Materials and Methods

This study conforms with the international ethical recommendations, and was conducted using non-invasive imaging approaches on the cadaveric forelimbs obtained from slaughtered horses intended to be used for public meat consumption. No animals were sacrificed to carry out this study. Additionally, this study did not receive any funding or grants from public, commercial or not-for-profit sectors or funding agencies.

The study was conducted using 29 *ex vivo* forelimbs, that were first diagnosed by radiography for the presence of the expected bone overgrowth exostosis of the P1, before being processed for subsequent investigations.

### Forelimbs

The forelimbs were obtained from Tbourida horses aged between 6 and 8 years and were slaughtered at the Rabat slaughterhouse in the north of Morocco (latitude: 34°01′ N, 6°50′ W) for public meat consumption. These horses originated from two neighboring regions (Khemisset, 33°49′ N, 6° 04′ W and Settat, 33°00′ N, 7° 37′ W). These horses were slaughtered because of emergency cases such as severe and untreated colics, limbs fractures and complicated laminitis with sole perforation. The *ex vivo* forelimbs of slaughtered horses are usually not consumed and were therefore generously donated by the slaughterhouse officials for research purposes.

Two to three Tbourida horses are slaughtered per week as the Rabat slaughterhouse. All recovered *ex vivo* distal forelimbs (*n* = 29), during the 3 months sampling period, underwent a radiographic investigation. Only six of these showed an exostosis of the P1 and were, therefore, included in this study.

**Figure 1:**
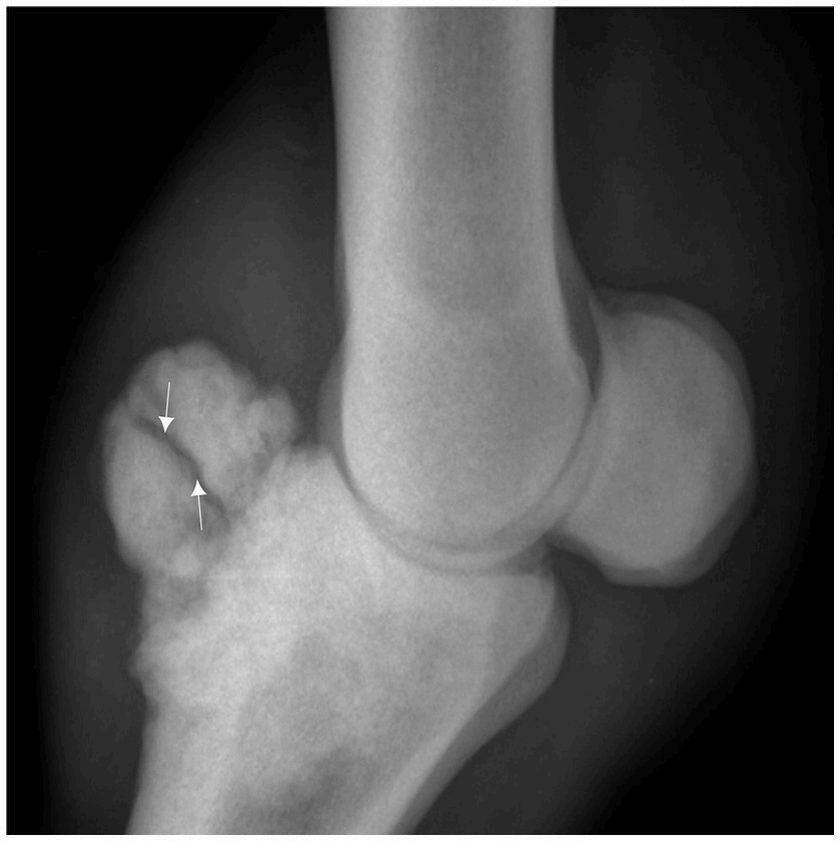
Lateromedial radiograph of the fetlock demonstrating a bone overgrowth exostosis on the dorsal and proximal part of the first phalanx in a 7 year old Arab Barb horse complicated with a fracture line (arrow).

Samples were cut at the mid canon, fixed in 10% neutral buffered formalin and stored at −4°C until subsequent use for gross anatomy description, CT, an MRI and histological analysis. The study was conducted in conformity with the Moroccan Ministry of Agriculture and the Hassan II Agronomy and Veterinary Institute of Rabat's recommendations, in accordance with international ethical standards (7). Radiography screening for two survey views (lateromedial and dorsopalmar), an X-ray generator (Medison Acoma® Co., Ltd., Tokyo, Japan) and a PROSCAN CR system for image development (Protec® GmbH Co. KG, Dorfwiesen, Germany), were employed. The radiographic constants were 60 KV with an exposure time of 0.25–0.3 s. The distance between the cassette and the X-ray generator was 70 cm. Only fetlocks that showed a bone overgrowth exostosis on the dorsal and proximal part of the P1 in radiographic exams were selected to be used in this study (Figure 1).

### Computed Tomography

The CT examination was performed from the MCPJ to the distal forelimb, using a system brand (Philips Brilliance CT®) with a 42-detector row CT scanner, in which limbs were placed in order to obtain transverse, sagittal and 3D reconstruction. Acquisition variables were 140 KV, 400 mA, 16 × 0.75 mm collimation, 0.4 mm for slice increments, a 0.75 s rotation time, a pitch of 0.313, a field of view of 200 mm and a matrix size of 768 × 768.

### Magnetic Resonance Imaging

The MRI was performed using a system brand (Hallmarq Standing® Equine MRI) with a permanent 0,27 T magnet and focused in the fetlock region. T1- and T2^*^-weighted gradient echo (GRE), T2 fast spin echo (FSE), and short tau inversion recovery (STIR) were acquired in the transverse, dorsal and sagittal planes. The slice thickness was 5 mm and the image matrix size was 170 × 170 with a field of view of 170 × 170 mm. The T1 GRE (echo time [TE] = 8 ms, Repetition Time [TR] = 52 ms), the T2^*^ GRE (TE = 13 ms, TR = 68 ms), the T2 FSE (TE = 88 ms, TR = 1544 ms), and the STIR FSE (TE = 22 ms, TR = 4,272 ms for dorsal plane and 3,204 ms for sagittal plane).

### Dissection

The dissection of the *ex vivo* fetlocks was performed based on the usual techniques of investigation in anatomopathological investigations (8). First, an evaluation of the lesion was performed by a simple inspection and palpation of the intact specimen. Afterward, based on the radiographic imaging, the skin was carefully incised around the lesion and then totally removed. The soft tissues surrounding the bone overgrowth exostosis, including the common and the lateral digital extensor tendons and the joint capsule, were inspected and dissected to be identified from the surrounding pathologic structures. The location of the lesion was determined by verifying possible compression consequences for surrounding vessels and nerves. The measure of the exostosis lesion included the largest diameter, length and width. Successive cross sections were made, in a proximal to distal direction, to investigate the deep extension of the lesion.

### Histology

After dissections, samples were obtained for histopathological investigations. Tissue samples included pieces from the bone overgrowth exostosis and the fetlock joint capsule. To ease obtaining microtome sections, the bone samples were first demineralized in a solution of EDTA disodium salt (Osteodec®, bio optica, Milano, Italy). They were then dehydrated, paraffin-embedded, cut into sections of 4 μm thicknesses using a microtome (Leica Microsystems®, Wetzlar, Germany) and finally stained with hematoxylin and eosin (H&E) and Masson's Trichrome, using classical histology procedures. The sections were viewed under a light microscope (Olympus BX40®) and images were taken with a connected camera (Nixon DS-Fi1). The Nikon NIS-Elements microscope imaging software was used.

## Results

### CT Findings

**Figure 2:**
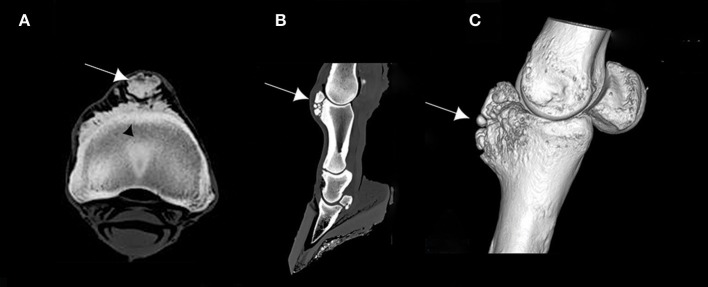
**(A)** Transverse computed tomography (CT) scan of the proximal part of the first phalanx (P1), **(B)** sagittal CT scan of the distal forelimb, and **(C)** 3D CT reconstruction of a right *ex vivo* metacarpophalangeal joint (MCPJ) of a Tbourida Horse, showing a bone remodeling of the proximal and dorsal aspect of the P1, with bone fragmentation (arrows). Cortical and medullary continuity of the bone overgrowth exostosis is clearly identified on **(A)** (black arrowhead).

**Figure 3:**
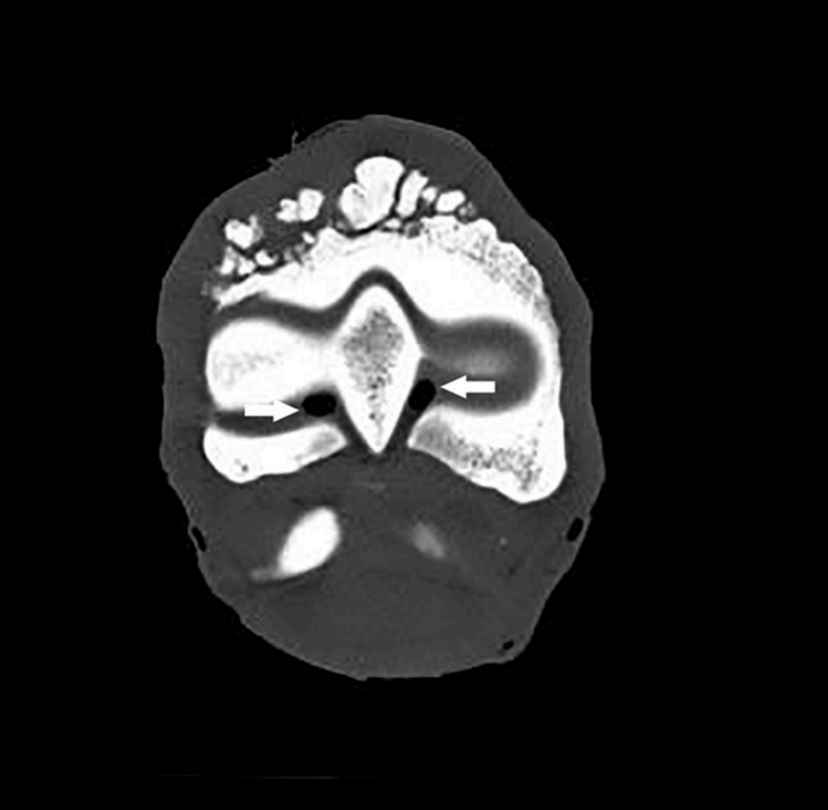
Transverse CT scan of the distal part of the third metacarpal bone showing a decrease of the radiopacity of the articular cartilage on each side of the sagittal ridge, which is consistent with a cartilage erosion of the MCPJ (arrows).

The CT images revealed bone remodeling on the dorsal and proximal surface of the P1, with the presence of several bone fragments at this level. At this stage, it was not yet clear if these fragments were included in the surrounding tissue. A cortical and medullary continuity of the overgrowth bone exostosis, with underlying bone, was clearly identified in the transverse reconstruction (Figure 2). The CT examination also revealed an early remodeling of the dorsodistal aspect of the third metacarpal bone (MCIII), at the attachment of the joint capsule in all *ex vivo* forelimbs. In addition, there was a decrease of the radiopacity of the articular cartilage on each side of the sagittal ridge of the MCIII (Figure 3). Other lesions were observed in different parts of the *ex vivo* forelimbs. They included ungular cartilage ossification (3/6 of cases) and bone remodeling of the dorsoproximal and dorsodistal part of the second phalanx (1/6 of cases).

### MRI Findings

**Figure 4:**
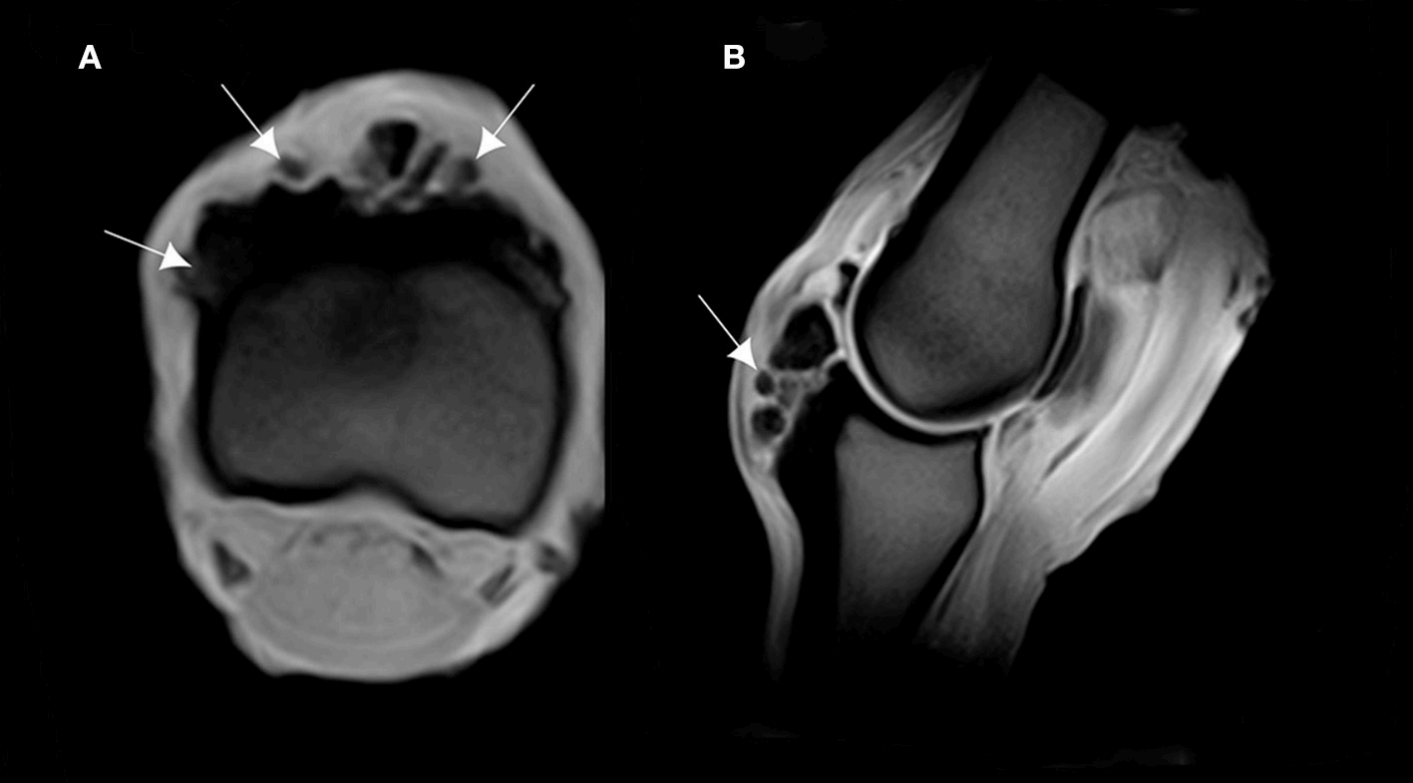
**(A)** T1-weighted gradient echo (GRE) transverse magnetic resonance imaging (MRI) of the P1, and **(B)** sagittal MRI of the MCPJ of an *ex vivo* forelimb of a Tbourida horse. Medial is to the right of the transverse image. The sagittal image corresponds to the lateral aspect of the MCPJ. An increase in signal intensity is observed on the dorsal and proximal part of the P1, which corresponds to the overgrowth bone exostosis (arrows).

**Figure 5:**
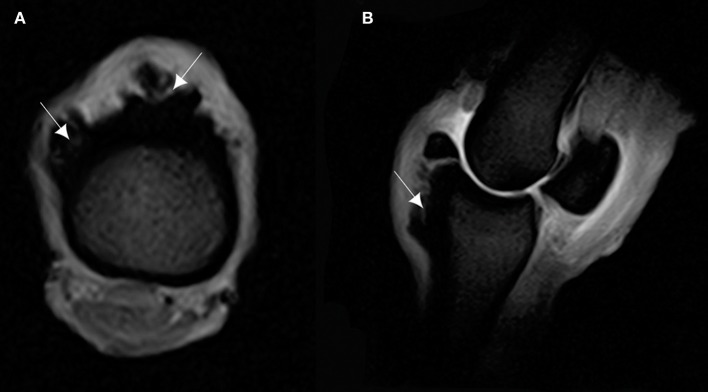
**(A)** T2* W GRE transverse of the proximal part of the P1, and **(B)** T2* W GRE sagittal images of a right MCPJ with MRI findings consistent with a cortical reaction of the dorsal and proximal part of the P1. The lesion is hyperintense in T2* W GRE images (arrows). Medial is to the right of transverse image. The sagittal image corresponds to the lateral aspect of the MCPJ.

**Figure 6:**
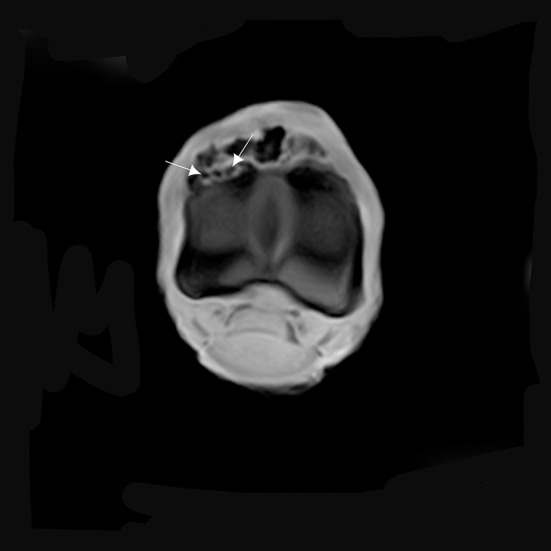
T1W GRE transverse image of the proximal part of the P1 with MRI findings revealing a continuity of the lesion with the marrow space of the affected bone (arrows). There is an increase in signal intensity at this level.

**Figure 7:**
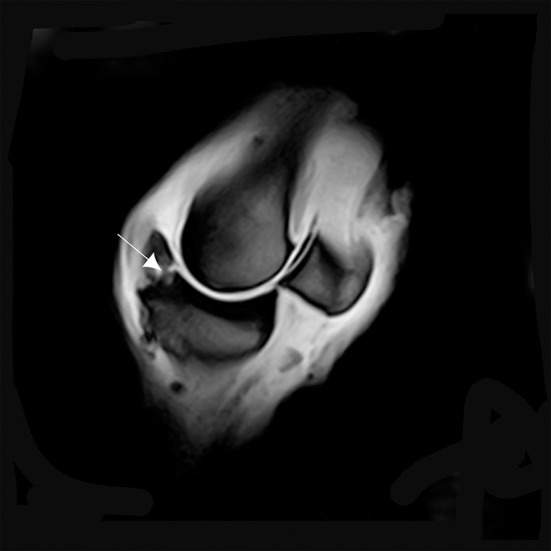
T1W GRE sagittal image of a left MCPJ with MRI findings consistent with osteochondrosis of the dorsal and proximal margin of the P1 (arrow). An increase in signal intensity at this level could be confused with a fracture line.

The MRI appearance of the bone overgrowth exostosis of the dorsal and proximal part of the P1, had an increase in signal intensity on T1W GRE FAST, T2^*^W GRE FAST, T2W FSE TRA FAST and STIR FSE SAG FAST. This lesion was well identified on the transverse and sagittal plane (Figure 4). A densification of the cortical bone of the P1 was characterized by an increase in signal intensity on T1W GRE FRO FAST, STIR FSE FRO FAST and T2^*^W GRE FAST. This reaction was observed in all cases and was most apparent on the lateral and proximal part of the P1 (Figure 5). Cross sectional imaging revealed a continuity of the lesion with the marrow space of the affected bone, as well as with the cortex at the lateral and medial sides (Figure 6). Five out of six cases showed a characteristic linear hyperintensity at the level of the lateral and medial eminences of the P1 on T1W GRE SAG FAST and T2^*^W GRE SAG FAST. This hyperintensity could be confused with a fracture line on the dorsal and proximal margin of the P1. This was well identified on the sagittal plane (Figure 7).

**Figure 8:**
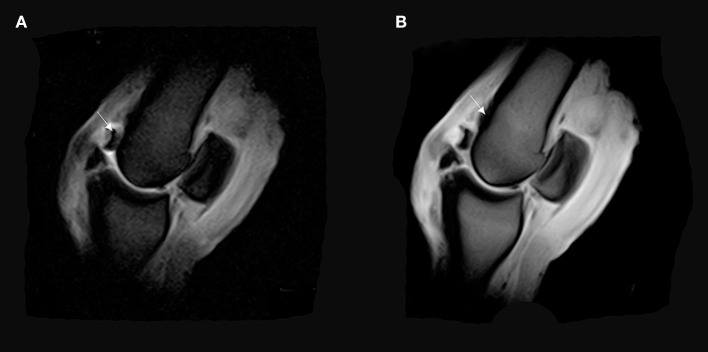
**(A)** T2^*^W GRE sagittal FAST image of a right MCPJ with a bone fragment revealing a possible osteochondrosis lesion on the dorsal and distal part of the MCIII (arrow). **(B)** T1 W GRE sagittal image of a right MCPJ. Evidence of an increase in signal intensity in the distal and dorsal part of the third metacarpal bone (MCIII). This signal pattern is consistent with a bone edema on the dorsal and proximal attachment of the fetlock joint capsule (arrow).

An increase in signal intensity in 3/6 of cases was also observed in the surrounding lesions at the distal and dorsal part of the MCIII. This signal pattern was consistent with a bone edema on the dorsal and proximal attachment of the fetlock joint capsule observed on T1W GRE SAG FAST. A bone fragment revealing a possible osteochondrosis lesion was observed on the dorsal and distal part of the MCIII and detected on T1W GRE FAST and T2^*^W GRE FAST. On the other planes, a densification of the cortical bone at this site was observed (Figure 8).

**Figure 9:**
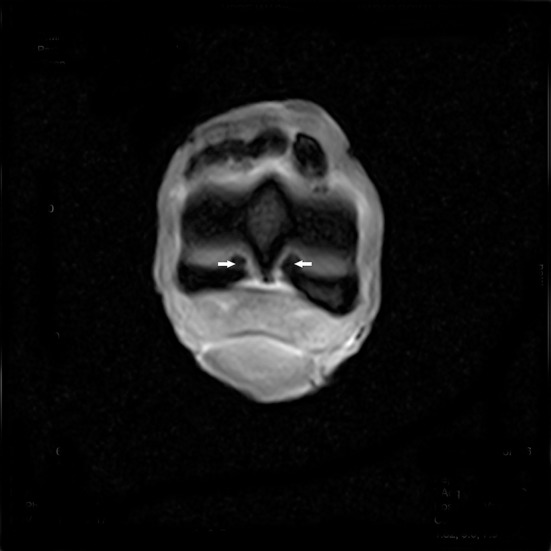
T2^*^W GRE FAST transverse image of the distal margin of the MCIII with hypointense signal pattern consistent with a cartilage erosion of the palmar aspect of the junction between the medial condyle and sagittal ridge, and between the lateral condyle and sagittal ridge of the MCIII (arrows).

Cartilage irregularities and hyposignal areas were detected on the cartilage of the distal part of the MCIII. This lesion, emanating from the synovial liquid to the subchondral bone of the MCIII, was observed on T1W GRE SAG FAST, T2^*^W GRE FAST, T2W FSE TRA FAST, STIR FSE FRO FAST, and STIR FSE SAG FAST. These hyposignal areas represent the cartilage erosion of the palmar aspect of the junction between the medial condyle and sagittal ridge, and between the lateral condyle and sagittal ridge of the MCIII (Figure 9). A focal decrease in the intensity was also observed on the dorsal aspect of the medial condyle of the MCIII.

### Gross Anatomical Lesions

**Figure 10:**
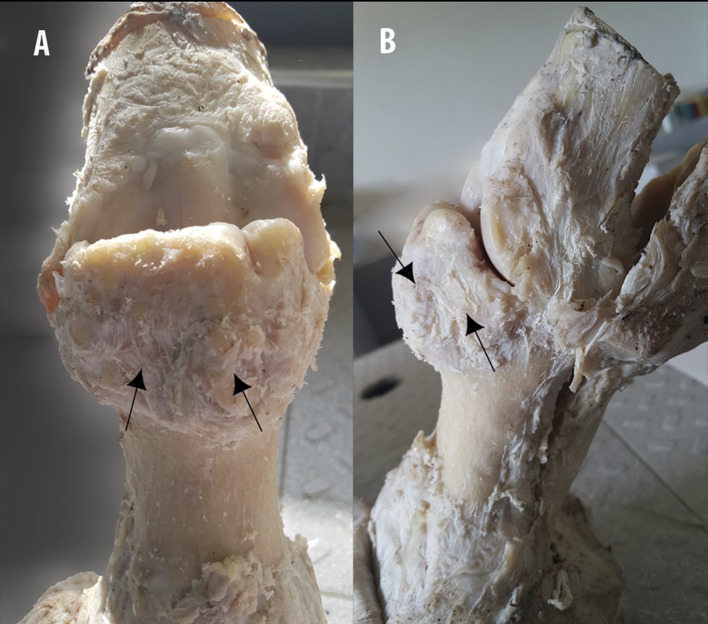
Gross examination and description on the fixed specimen. **(A)** Dorsopalmar and **(B)** lateromedial aspects of a MCPJ after careful dissection, demonstrates the aspect of the bone overgrowth exostosis on the dorsal and proximal part of the P1, showing poly-lobulated masses of tissue with a cauliflower shape (arrows).

**Figure 11:**
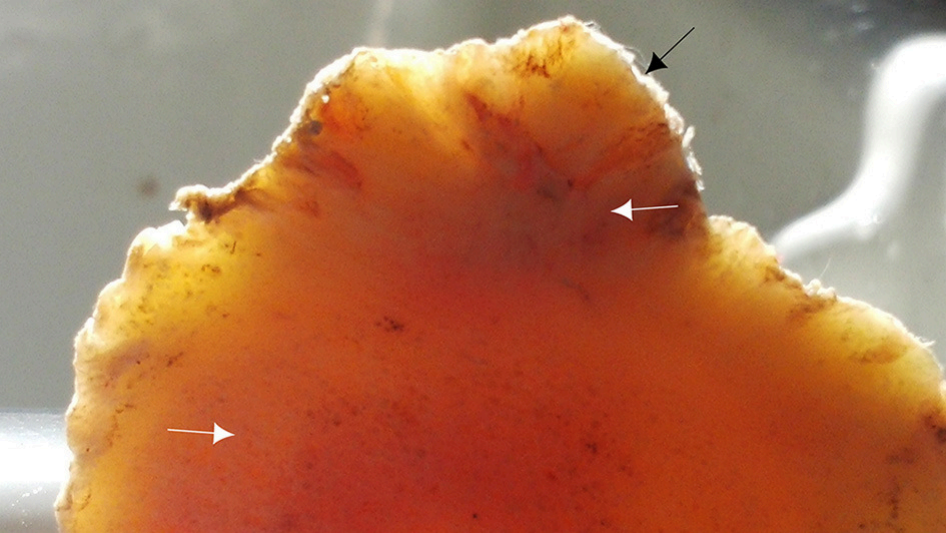
Transverse section of the proximal part of the P1 showing irregularities of the dorsal aspect of the P1 (black arrow) and the continuity of the lesion with cancellous bone of the marrow space (white arrows).

After careful dissection and separation of the skin and soft tissue surrounding the dorsal part of the fetlock joint (common and lateral digital extensor tendons), the bone overgrowth exostosis appeared as a mass arising from the surface of the bone. The lesion was clearly identified on the dorsal and proximal side of the P1 (Figure 10). The shape and the size were different from one limb to another. The diameter/depth varied from 0.5 to 5.1 cm with a mean of 3.9 ± 0.9 cm. This bone lesion involved almost all the dorsal and proximal parts of the P1, but the diameter was much larger on the lateral side. The surface was irregular with poly-lobulated tissue masses showing a cauliflower shape corresponding to ossification centers. The lesion was continuous with cancellous bone surrounding the marrow space of the parent bone (Figure 11). A cap of irregular cartilaginous tissue covered the surface.

**Figure 12:**
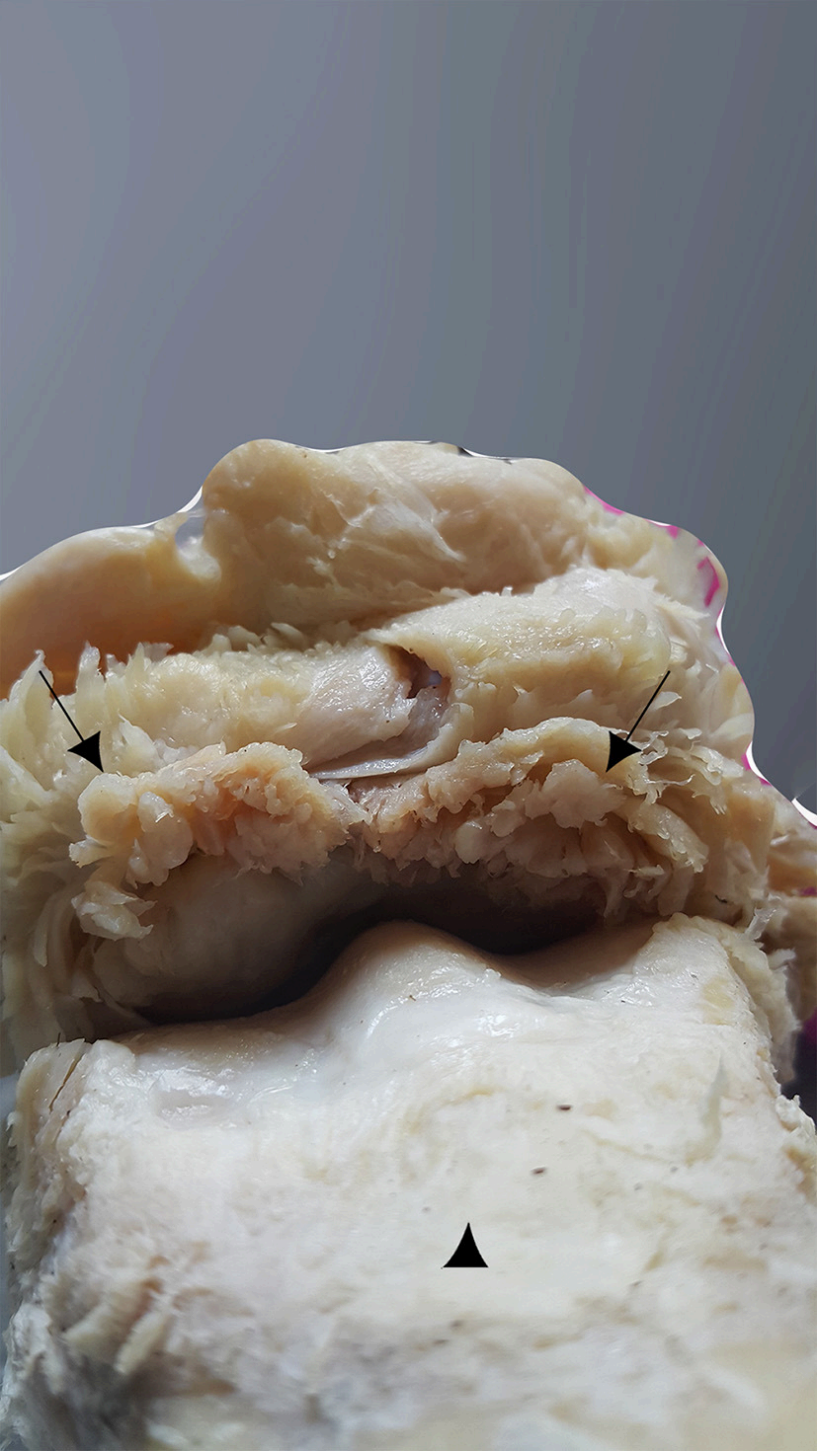
Dorsal aspect of the MCPJ after careful dissection, showing a hypertrophic capsule joint on its dorsal side and many invaginations on its inner part, which is in contact with the bone overgrowth exostosis (black arrows). The MCIII is at the bottom of the image (arrowhead).

The dorsal part of the fetlock joint capsule was hypertrophic and revealed many invaginations in the inner part, in contact with the bone overgrowth exostosis (Figure 12). The lesion also showed clear compression and deformities of the surrounding structures especially the skin, the common and lateral digital extensor tendons, the extensor branches of suspensory ligament, and neurovascular bundles.

### Histological Findings

The histopathological examination of the bone overgrowth exostosis lesions on the P1, revealed an extensive proliferation of hyaline cartilage forming a cap, including large foci of endochondral ossification, and cartilage canal vessels, with a base of cancellous bone surrounding marrow spaces. These histological findings are in accordance with the major criteria of osteochondroma diagnosis.

**Figure 13:**
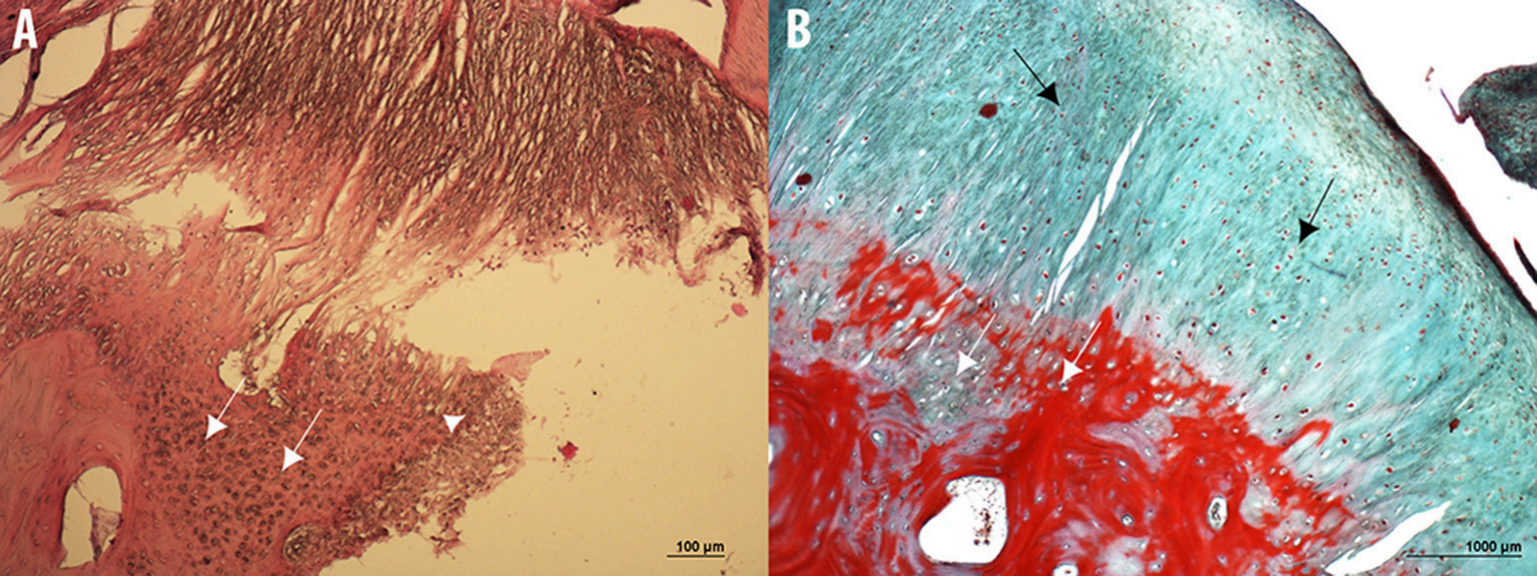
Microphotographs of hematoxylin & eosin **(A)** and Masson's Trichrome **(B)** stained sections of the bone overgrowth exostosis of the P1 from a 5 year old Tbourida horse, revealing an extensive proliferation of hyaline cartilage forming a cap, including a large foci of endochondral ossification and cartilage canal vessels (white arrows), with a base of cancellous bone surrounding the marrow spaces. Limited areas of necrosis and cystic vacuolation surrounded by nuclei of cell proliferation can also be observed (white arrowhead on image **A**), with a fibrous capsule or perichondrium (black arrows in image **B**). These histological findings confirmed the diagnosis of an osteochondroma on the P1.

Histopathological analysis also showed limited areas of necrosis and cystic vacuolation surrounded by nuclei of cell proliferation (Figure 13).

At the most proximal part of the lesion and adjacent to the articular space of the fetlock joint, other types of lesions were detected, represented by a limited area of necrosis with formed capillary blood vessels (revascularization) which were observed with granulation tissues accompanied by osteoclast proliferation with resorption of necrotic bone tissue, and newly formed lamellar bone tissue. This histopathological finding suggests the diagnosis of an osteochondrosis.

**Figure 14:**
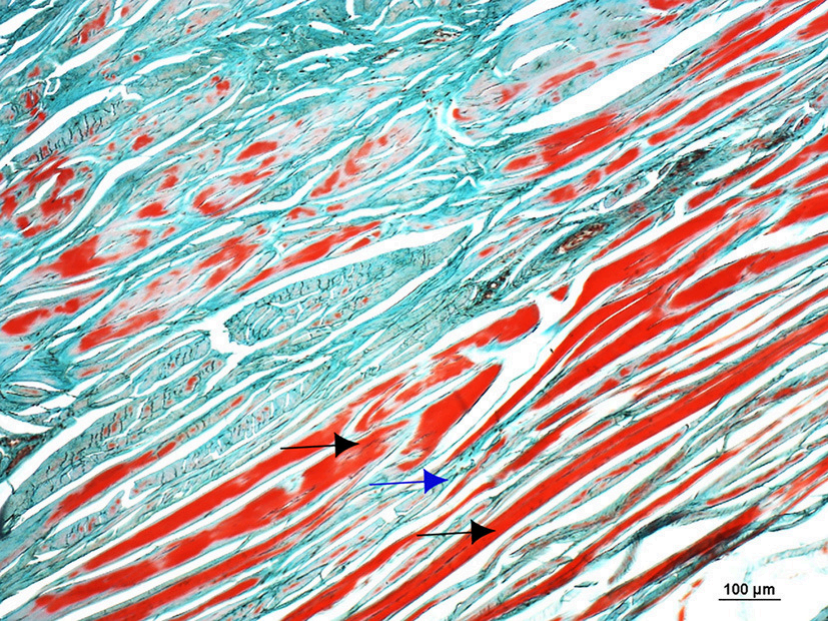
Microphotograph of a Masson's Trichromestained section of the dorsal and proximal aspect of the joint capsule of the fetlock, showing a large amount of recently formed connective tissue fibers in the inner part of the capsule (black arrows) interspersed with mature connective tissue (blue arrow). The connective tissue fibers show a non-regular and non-straight orientation of the main axis.

As in the gross anatomy description, the articular capsule showed extensive lesions, revealed both by H&E and Masson's Trichrome histological staining's. A large amount of recently formed connective tissue fibers, in the inner part of the capsule interspersed with mature connective tissue, was observed. The connective tissue fibers showed a non-regular and non-straight orientation of the main axis, and large amounts of newly formed capillary blood vessels (revascularization) in focal areas were also observed (Figure 14).

## Discussion

This study was conducted to characterize the anatomo-histopathological and the imaging features of the MCPJ on *ex vivo* forelimbs of Tbourida horses, presenting a particular bone overgrowth exostosis previously diagnosed with an X-ray exam.

Several pathological processes have been reported to induce bone or cartilage exostosis for both humans and animals, especially in horses and dogs. These mostly include osteochondroma, periosteal chondroma (also known as juxta cortical chondroma), and enchondroma (9, 10).

Based on the results of this study, using different methods of investigation including radiography, CT, an MRI, gross anatomy and histopathology, the lesions observed correspond to an osteochondroma of the dorsoproximal part of the P1. Histologically, the main findings were the presence of an extensive proliferation of cartilage, including large foci of endochondral ossification and cartilage vessels surrounding cancellous bone, which is consistent with the microscopic features of an osteochondroma, as previously described in horses (9).

The main criteria leading to the diagnosis of the osteochondroma on the CT scan, is the presence of cortical and medullary continuity with the underlying bone; cross sectional imaging is useful for confirming this continuity (10). An MRI is also necessary to confirm the continuity between the osteochondroma and the cortex of affected bone. Additionally, an MRI could differentiate an osteochondroma from other surface bone lesions, and usually shows a high signal on the T2-weighted scan of the cartilaginous cap of the lesion and a low one on the T1-weighted scan (11). Histologically, osteochondroma is described as an apical margin of hyaline cartilage with a base of cancellous bone, surrounding marrow spaces (12).

In horses, the most common location for osteochondroma is the distal and caudal part of the radius (13–18). The same lesion was also reported on the middle phalanx (19), calcaneus (20), and caudal distal tibia (21, 22). Osteochondroma was also described in an 18-months-old warm blood clot in nasal bone (23). Osteochondroma is defined as a benign cartilage-capped exostosis arising from the surface of bones, formed by endochondral ossification adjacent to the physis or subarticular growth plate (12, 24). Malignant transformation to chondrosarcoma or osteosarcoma has been reported in dogs and humans but not in horses (12). Osteochondroma is considered as a manifestation of a dysplastic physeal growth rather than a true neoplasm (12, 24). It is thought to result from the separation of a portion of the metaphyseal growth plate margin, creating an island of chondrogenic tissue, capable of endochondral ossification that is carried into the metaphysis with the growth of bone, resulting in a cartilage capped exostosis projecting from the metaphyseal surface. This growth is said to stop at skeletal maturity (12). In mature osteochondroma, endochondral ossification may be complete, and little or no cartilage may remain at the apical surface (12).

Strong arguments are in favor of the possible presence of this osteochondroma at an early age of Tbourida horses. Indeed, in our previous radiographic study, the bone overgrowth exostosis of the dorsal and proximal part of the P1 was diagnosed in different age groups, including 2 years old. This is in accordance with lesions described in human teenagers (25, 26). The lesion arises from the separation of a portion of tissue from the metaphyseal growth plate of the P1 during skeletal growth. Indeed, osteochondroma was described in patients in their second decade of life and more commonly in phalanges than metacarpals (25, 26). As in Tbourida horses, the lesion in humans is also observed in the lateral side. It results from a displacement of the lateral portion of the growth plate in a diagonal direction of the long axis of the tubular bone and away from the articular joint (11).

Osteochondroma in horses should be differentiated from other bone overgrowth exostosis on the P1, such as the degenerative joint disease of the proximal interphalangeal joint also known as “high ringbones” and which especially affects the distal part of the P1 and the proximal part of the second phalange, causing lameness. Traumatic accidents, arthritis, osteochondrosis, poor limb conformation, and repeated stress to periarticular fibrous tissue structures are responsible for the onset of this lesion (27).

Periosteal chondroma, also known as juxta cortical chondroma, is a benign neoplasm described in humans and some animals and should be differentiated from osteochondroma (10). This lesion arises from the periosteum or the cortex on the bone surface, without involvement of the medullary cavity (28). In horses, chondroma was described in the arytenoid cartilage (29). Radiographically, periosteal chondroma is described as a lucent surface with a scalloping of the underlying cortex. On an MRI, high signal intensity on the T2-weighted scan, reflecting the cartilaginous consistency of the lesion, has been described (11).

In this study, different lesions, adjacent to the osteochondroma, were observed in the examined forelimbs. This included capsulitis associated with a large amount of recently formed connective tissue fibers in the inner part of the capsule fetlock joint. This lesion can be associated with joint damage and chronic injections (30). In the present study, the observed osteochondroma on the dorsal aspect of P1 and its continuous trauma on the joint capsule, are likely involved in the appearance of capsulitis. Osteochondroma itself could also be a consequence of a direct external trauma. Indeed, previous trauma or surgery in the fetlock joint could induce a solitary osteochondroma (31). Small dislodged cartilage parts may become trapped in the synovium, develop a blood supply and then grow to form osseous masses. The commonly applied hobbles around the pastern joint in stabled Tbourida horses may be involved in the etiology of both capsulitis and osteochondroma.

Hyperextension of the fetlock joint in the horse may result in a partial tearing of the joint structures and can induce the formation of exostosis (32). Such hyperextensions at maximal weight-bearing during racing speed or upon landing after a jump leads the MCIII to reach a right angle. Indeed, at such full stance the dorsal surface of the P1 is compressed by the dorsal articular surface of the MCIII, producing compression and shear (33). In Tbourida horses, during the sudden break after a fast gallop, the forelimb fetlock joint undergoes a hyperextension, causing repetitive sliding of the common and lateral digital extensor tendons on the P1, and its contact with the distal aspect of the MCIII induces successive traumas and the appearance of an osteochondroma lesion in the P1.

In addition, the biomechanical effect of the ground surface leads to musculoskeletal injuries in horses. It was previously demonstrated that a comfortable track compared to a hard track, reduces the amplitude of the shock at hoof impact (about 50%), decreases the generated vibrations and produces a more gradual deceleration during the braking phase of the hoof (34). Tbourida shows usually take place on a hard surface. The shock waves generated at the hoof are intense and certainly affect the osteoarticular and the soft tissue of the distal forelimb parts. Combined with the trauma caused by the hyperextension of fetlock joint, as discussed above, these shocks surely cause repetitive trauma producing a separation of a portion of the metaphyseal growth plate margin of P1, creating an island of chondrogenic tissue which induces an osteochondroma.

The revealed high incidence (6/29) of this pathology in this study could be related to the age of the slaughtered horses (between 6 and 8 years old). This age interval corresponds to a period of high physical activity and competition services and, therefore, horses are predisposed to endure fractures in different parts of the forelimbs. In most cases, Tbourida horses are sacrificed for an emergency-slaughter that is related to fractures but also for severe and untreated colics and complicated laminitis with sole perforation. In fact, the traditional management practices are incriminated in all these severe pathologies. Compared to other equestrian sport horses, Tbourida horses are known for their poor limb conformation and a poor farriery material. Feeding management also causes serious consequences. Despite the lack of literature about Tbourida horses, it is well known locally that these horses are fed uncontrolled quantities of concentrate. The owner's objective is to obtain fat horses, considered a criteria of beauty, in Tbourida shows. This high energy food intake induces a high frequency of laminitis, often complicated with sole perforation.

Overall, in Tbourida horse, we think that traditional management practices, like the use of hobbles during horse stabling and dietary imbalance associated with a poor limb conformation with a typical position of the horse during Tbourida show (exhibiting a hyperextended fetlock joint) especially at the sudden break, demanding repetitive sliding of the common and lateral digital extensor tendons on the P1 and a hard surface ground during the show may be responsible for a high frequency of osteochondroma and eventually its fracture.

## Conclusion

This study revealed the presence of osteochondroma of the P1 in Tbourida horses. To the best of our knowledge, this is the first report of an osteochondroma in the P1 of horses. Moreover, this is the first study on such lesions, using different techniques including: radiography, CT scans, an MRI, gross anatomy and histopathology. The detection in our study of a cartilaginous cap on the cancellous bone suggests that the endochondral ossification was not complete. An awareness campaign should be undertaken with owners of Tbourida horses, to overcome the inadequacy of dietary imbalance, poor farriery and poor limb conformation. The surface ground should be managed and made more comfortable for Tbourida shows, by using quality sand such as that used for racetracks of thoroughbred horses.

## Author Contributions

MS, RA, and KE contributed to the study design, prepared the specific aims and methods, compiled the data, prepared the manuscript draft and discussed the results and conclusions. EB performed the histopathological analyses. MP, HA, HB, and JC participated in the discussions and edited the manuscript draft.

### Conflict of Interest Statement

The authors declare that the research was conducted in the absence of any commercial or financial relationships that could be construed as a potential conflict of interest.
